# Long-term prophylaxis with lanadelumab for HAE: authorization for temporary use in France

**DOI:** 10.1186/s13223-022-00664-4

**Published:** 2022-04-01

**Authors:** Olivier Fain, Aurelie Du-Thanh, Delphine Gobert, David Launay, Neil Inhaber, Karima Boudjemia, Magali Aubineau, Alain Sobel, Isabelle Boccon-Gibod, Laurence Weiss, Laurence Bouillet

**Affiliations:** 1 service de médecine interne, AP-HP, Hôpital Saint Antoine, Sorbonne Université, 75012 Paris, France; 2 Dermatology Department, Centre Hospitalier Universitaire de Montpellier, Montpellier, France; 3 U1286—Infinite—Institute for Translational Research in Inflammation, Department of Internal Medicine and Clinical Immunology, National Reference Centre for Angioedema (CREAK), Inserm, CHU Lille, University of Lille, Lille, France; 4 Takeda Development Center Americas, Inc, Lexington, MA USA; 5 Takeda Pharmaceuticals International AG, Paris, France; 6 Department of Internal Medicine, CHU Edouard Herriot, Lyon, France; 7 Hotel-Dieu de Paris, Université de Paris, Paris, France; 8 Department of Internal Medicine, National Reference Centre for Angioedema (CREAK), INSERM-CNRS-CEA, CHU Grenoble, Université Grenoble Alpes, Joint Unit, 1036 Grenoble, France

**Keywords:** Authorization for temporary use, France, Hereditary angioedema, Lanadelumab

## Abstract

**Background:**

Hereditary angioedema (HAE) is associated with a heavy burden of illness.

**Objective:**

To evaluate use of lanadelumab in a French Authorization for Temporary Use (ATU) program.

**Methods:**

ATU requests were made between October 12, 2018, and March 13, 2019; patients were followed through September 23, 2019. At entry, patients received lanadelumab 300 mg every 2 weeks. HAE attack characteristics were evaluated at day (D) 0 and months (M) 3 and 6. Patients completed the Angioedema Quality of Life (AE-QoL) questionnaire at initiation and monthly and the Angioedema Activity Score questionnaire daily in 28 day cycles (AAS28).

**Results:**

In total, 77 patients received ≥ 1 lanadelumab dose; 69 had ≥ 1 quarterly follow-up visit (analyzed population). Mean (standard deviation [SD]) lanadelumab exposure was 240.4 (53.7) days. Lanadelumab dose was modified in 12 patients (mostly to every 4 weeks). For the analyzed population, compared with attacks/month (mean [SD]) within 6 months before ATU (2.68 [2.54]), fewer attacks occurred between initiation and first visit (0.16 [0.42]; P < 0.001) or last visit (0.16 [0.42]; P < 0.001); D15 and last visit (0.15 [0.41]); and D70 and last visit (0.17 [0.70]). AE-QoL total and domain scores were significantly higher at initiation versus M3 and M6; 55% and 65% of patients, respectively, achieved a minimal clinically important difference from D0 to M3 and D0 to M6. Proportion of patients with AAS28 of 0 was higher during M3 (90%) and M6 (83%) than initiation (59%). The most frequently reported adverse events included headache (7.3%) and injection site pain (6.3%).

**Conclusions:**

Lanadelumab reduced attack rates, improved quality of life, and was generally well tolerated.

**Supplementary Information:**

The online version contains supplementary material available at 10.1186/s13223-022-00664-4.

## Introduction

Hereditary angioedema (HAE) is a rare disease associated with C1 inhibitor (C1-INH) deficiency or dysfunction caused by *SERPING 1* mutations (HAE type 1 or 2 [HAE-1/2]), resulting in recurrent swelling episodes affecting subcutaneous or submucosal tissues [1–3]. Occurrence of HAE in patients with normal C1-INH levels is increasingly reported; several genetic mutations have been identified (e.g., *Factor XII*, *plasminogen*, *Angiopoietin-1*, and *kininogen-1*), although the underlying cause often remains unknown [2, 4, 5].

HAE attacks fluctuate throughout life with unpredictable frequency, severity, and duration, leading to wide-reaching physical, social, and psychological effects that negatively impact patients’ daily lives, even during symptom-free periods [1, 6, 7]. Given the heavy burden of disease and the episodic, unpredictable nature of attacks, long-term prophylaxis (LTP) is an important consideration for many patients [8]. Due to recurrent shortages of plasma-derived C1-INH in 2017 and 2018, the French Reference Centre for Angioedema (CREAK) issued recommendations that all available treatments be used for LTP, including use that was off-label, to ensure adequate patient management.

Lanadelumab is a fully human monoclonal antibody inhibitor of plasma kallikrein that is approved in several countries and regions for the prevention of attacks in patients aged ≥ 12 years with HAE [9, 10]. Current HAE treatment guidelines recommend lanadelumab as a first-line LTP option for patients with HAE-1/2 [2, 11]. Efficacy and safety of lanadelumab in preventing HAE attacks was demonstrated in the HELP Study (NCT02586805) [12] and HELP open-label extension (OLE; NCT02741596).

An Authorization for Temporary Use (ATU) is a compassionate early-access program allowing for advanced use of a medication that has not yet received marketing authorization; in France, criteria defined in the French Public Health Code must be met [13]. In particular, an ATU allows for advanced use of a medication that meets the following key criteria: efficacy and safety are strongly presumed; intended to treat serious or rare diseases when no appropriate treatment exists and the initiation of treatment cannot be deferred; and marketing authorization has not yet been granted [13]. On August 29, 2018 (before the approval of lanadelumab in the European Union), the French National Agency for Medicines and Health Products Safety (Agence Nationale de Sécurité du Médicament et des produits de santé [ANSM]) granted an ATU in a cohort (cATU) to Shire, a Takeda company, for the use of lanadelumab in the routine prevention of attacks in patients aged ≥ 12 years with HAE-1/2, for whom other treatments used for routine prevention were ineffective or unavailable. Findings for patients participating in this program are presented herein.

## Methods

ATU requests were made between October 12, 2018 and March 13, 2019; eligible patients were followed through September 23, 2019 (per ASNM protocol, Shire, a Takeda company, was required to submit a summary report of findings every 6 months). Of note, patients continued to the post-ATU phase once the initial ATU was completed; as mentioned later in the Discussion, the SERENITI study is underway in France.

In line with local regulations, neither approval from a local ethics committee nor written informed consent were required. However, the ATU protocol was subject to approval by the ANSM before initiation. Also, before seeking treatment access, physicians obtained verbal consent from each patient for treatment with lanadelumab, which could be done via telephone.

### Flow of communications

Participation in this program required organized communication between Shire (a Takeda company), the prescribing hospital physician, the dispensing hospital pharmacist, and the patient. If a patient did not meet the eligibility criteria for participation in the cATU (age ≥ 12 years with HAE-1/2, other treatments used for routine prevention were ineffective or unavailable for the patient). The prescribing physician could request a nominative ATU (nATU). Decisions regarding granting approval for nATU entry were made by the ANSM. Patient data were collected, processed, and stored as per the French Informatics and Liberties Law.

### Administration schedule

At ATU entry, all patients were prescribed subcutaneous lanadelumab 300 mg every 2 weeks. Dosage adjustments could be made at physician discretion after ≥ 6 months of treatment. Pharmacists dispensed lanadelumab on a monthly basis, as prescribed by the physicians.

### Effectiveness assessments and timing

The patient and disease history were collected from the patient’s chart, including the number of attacks reported during the 6 months prior to initiating lanadelumab. Other clinical data were prospectively collected during follow-up visits at the time of regular routine visits (estimated to be every 3 months but conducted per usual practice). Patient-reported outcome measures including Angioedema Activity Score (AAS) (daily over 4 weeks) and Angioedema Quality of Life (AE-Qol) questionnaire (once covering the last 4 weeks) were collected prospectively in a diary just before each visit. AE-Qol also was assessed immediately prior to lanadelumab initiation. Patients provided information in the diary on each injection at each injection time. All HAE attacks were physician confirmed. Characteristics of attacks (including frequency, type, location, and severity) and adverse events (AEs) were collected at the time of treatment access request, treatment initiation, and every 3 months during follow-up visits; the schedule for assessments is shown in Additional file 1: Table S1. Patients receiving LTP at the time of the treatment access request were required to discontinue treatment prior to lanadelumab initiation; however, no washout period was required.

### Quality of life assessments

The validated AE-QoL questionnaire [14] was completed at treatment initiation and monthly thereafter; data were collected during follow-up visits. The AE-QoL questionnaire is comprised of 17 items that are used to calculate four domain scores (functioning, fatigue/mood, fears/shame, and nutrition) and a total score. Scores are based on a 4 week recall period; lower scores reflect less impairment in health-related quality of life (QoL) [14]. The minimal clinically important difference (MCID) for the AE-QoL total score is 6 points (i.e., a decrease of ≥ 6 points signifies a clinically significant QoL improvement) [15].

AAS questionnaires were completed daily in 28 day cycles (AAS28), and findings were collected at follow-up visits. The validated AAS questionnaire assesses disease severity and burden associated with recurrent angioedema. Patients indicated whether an angioedema attack occurred in the previous 24 h [16–18]; lower AAS28 scores reflect lower disease activity [16, 17]. An AAS28 score of 0 denotes that no attacks occurred during the 28 day period, whereas scores > 0 indicate occurrence of ≥ 1 attack during this time. The minimum and maximum possible AAS28 scores are 0 and 420, respectively [18].

### Statistical analyses

The cumulative proportion of attack-free patients was calculated based on Kaplan–Meier estimates. Monthly HAE attack rate differences between the 6 months before ATU entry and after lanadelumab initiation were compared using the paired Wilcoxon signed-rank test.

Evolution of the final mean AE-QoL score and scores of the four dimensions over time were analyzed via a two-factor linear mixed model (at day [D] 0 and months 3 and 6). Occurrence of attacks after D0 (yes/no) was included as a covariate in this analysis to assess whether this had an impact on change in AE-QoL scores.

Baseline characteristics were compared between subgroups of patients with or without C1-INH LTP at ATU entry using the χ^2^ test or Student’s *t*-test in case of normal distribution or the Fisher test or Wilcoxon test otherwise.

A multivariate logistic regression analysis was conducted to identify factors associated with occurrence of all attacks, as well as those of treated HAE attacks from D0, D15, and D70. This analysis also was conducted to evaluate factors associated with AE-QoL scores that met the MCID (yes/no). Explanatory variables assessed for both analyses are shown in Additional file 2: Table S2.

### Exploratory analysis: lanadelumab effectiveness based on ongoing C1-INH LTP use at ATU entry

Lanadelumab effectiveness and impact on patient QoL were evaluated and compared between two patient subgroups based on whether they were receiving LTP with C1-INH agents at ATU entry (including intravenous [IV] plasma-derived [pd] C1-INH concentrate, fixed dose; IV pdC1-INH concentrate, weight based; or IV recombinant C1-INH concentrate).

### Exploratory analysis: patients with follow-up duration above the median

The planned duration between follow-up visits was every 3 months; however, the potential for variability in follow-up duration between lanadelumab initiation (D0) and visits 1 or 2 was anticipated. To obtain a patient sample with less variability, effectiveness of lanadelumab (measured by mean [standard deviation (SD)] and median [range] monthly rate of HAE attacks) was evaluated in a subgroup of the analyzed population with a follow-up duration above the median.

### Injection training and satisfaction

At D0, patients were asked to evaluate the ease with which they learned injection technique, based on a scale from 1 (very difficult) to 10 (very easy). Thereafter, patients completed injection use satisfaction questionnaires after each administration, using a scale from 1 (not at all satisfied) to 10 (very satisfied).

## Results

### Patient disposition and lanadelumab exposure

**Fig. 1:**
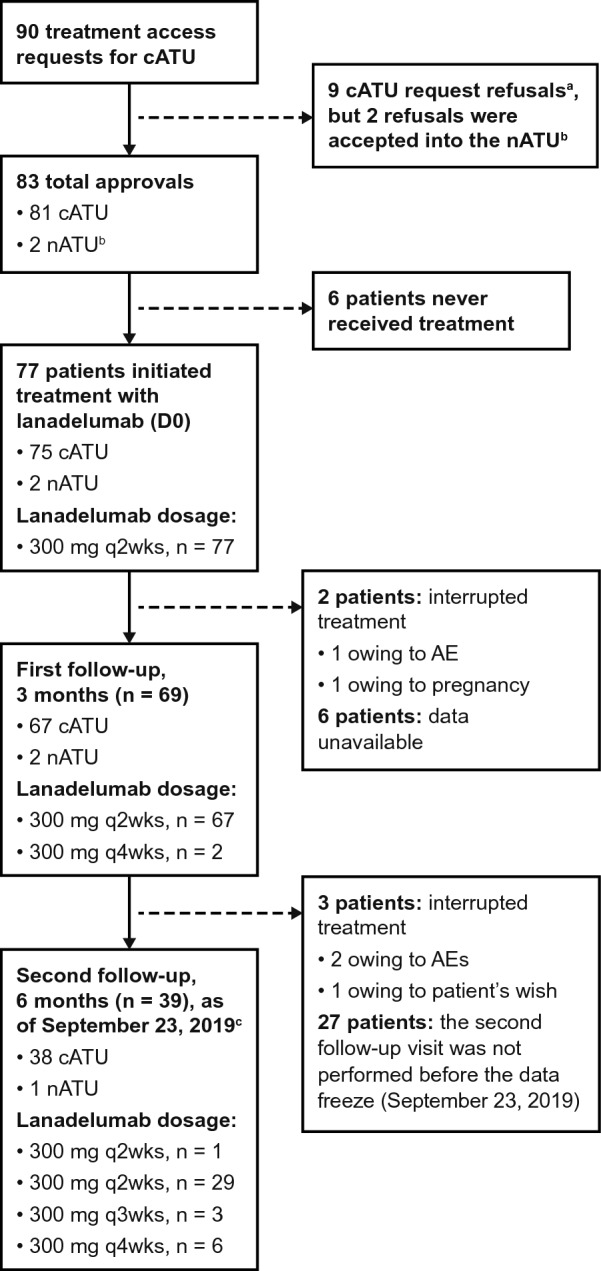
Patient disposition. ^a^Reasons for refusal for seven patients: incomplete information on hereditary angioedema (HAE) attacks 6 months before access request (n = 3); stable with no recent attacks (n = 2); insufficient patient information (n = 1); acquired angioedema (n = 1). ^b^One patient with acquired angioedema; one patient (with HAE-1) aged < 12 years. ^c^Lanadelumab was initiated (D0) on average ~ 46.5 days after the treatment access request; and follow-ups were counted from D0, not from Authorization for Temporary Use (ATU) entry. By the September 23, 2019, data freeze, not all patients had reached the 6 month follow-up period, or data for these patients were incomplete. *AE* adverse event, *cATU* ATU in a cohort*, D* day, *nATU* nominative ATU, *q2wks* every 2 weeks, *q3wks* every 3 weeks, *q4wks* every 4 weeks

Of 90 cATU requests received, 81 (90%) were approved. Of the nine requests denied, two nATUs were granted (one patient with acquired angioedema; one patient aged < 12 years with HAE-1). Thus, 83 patients were accepted into the ATU, 77 of whom received ≥ 1 dose of lanadelumab (75 as part of the cATU and 2 as part of the nATU; Fig. 1). Of these, 69 patients had ≥ 1 quarterly follow-up visit, constituting the analyzed population. As of September 23, 2019 (the end of the data collection period), patients were exposed to lanadelumab for a mean (SD) of 240.4 (53.7) days (median [range] 235.0 [80.0–335.0] days).

### Patient population

Baseline demographics and characteritics of the total population (n = 77) who received ≥ 1 lanadelumab dose are shown in Table 1. Enrolled patients were primarily female (69%), had HAE-1 (88%), and were aged ≥ 18 to < 65 years (82%); median age was 42.4 years.

**Table 1:** Baseline demographics at time of treatment access request for the total population who received ≥ 1 lanadelumab dose

Patient characteristic	n = 77
Median (range) age, y	42.4 (11.7–78.9)
Age, y, n (%)	
< 18	6 (7.8)
18–64	63 (81.8)
≥ 65	8 (10.4)
Female, n (%)	53 (68.8)
Weight (kg)	
Mean (SD)	75.8 (20.6)
Age at diagnosis, years	
Median (range)	10.0 (1–48)
HAE type	
1	68 (88.3)
2	7 (9.1)
HAE nC1-INH^a^	1 (1.3)
Acquired angioedema	1 (1.3)
Positive family history of HAE, n (%)	52 (67.5%)
Proportion of laryngeal attacks for the 3 most severe attacks in the 6 months prior to ATU entry, n (%)	
Number of severe attacks	149
Proportion of pharyngo-laryngeal attacks	12 (8.1)
Number of HAE attacks in the 6 months prior to ATU entry, median (range) (n = 70)	13.5 (1–99)
Exposure to LTP prior to ATU entry, n (%)^b^	72 (93.5)
Ongoing LTP prior to ATU entry, n (%)	60 (77.9)
Single oral agent^c^	20 (26.0)
Single C1-INH agent^d^	23 (29.9)
Combination of LTP agents	17 (22.1)
Monthly attack rate 6 months prior to ATU entry for patients with ongoing LTP, mean (SD)	n = 52 2.53 (2.67)
Number of attacks in the 6 months prior to ATU entry by ongoing LTP mean (SD)	
No LTP	n = 13 20.0 (8.8)
Single oral LTPs	n = 18 12.9 (8.7)
Single C1-INH LTPs	n = 17 17.4 (13.3)
LTP combinations	n = 17 15.4 (23.4)

*ATU* authorization for temporary use, *C1-INH* C1 inhibitor, *HAE* hereditary angioedema, *IV* intravenous, *LTP* long-term prophylaxis, *HAE nC1-INH* HAE with normal C1-INH levels, *pdC1-INH* plasma-derived C1-INH, *SD* standard deviation

^a^One patient with HAE with normal C1-INH levels (HAE Type III) was erroneously granted treatment access

^b^Refers to exposure at any time during the patient’s life

^c^Oral agents included danazol (n = 11), tranexamic acid (n = 3), progestins (n = 5), and rituximab (n = 1 [the nominative ATU patient with acquired angioedema]); 3 patients were receiving C1-INH in combination with danazol

^d^IV pdC1-INH concentrate, fixed dose (n = 11); IV pdC1-INH concentrate, weight based (n = 4); IV recombinant C1-INH concentrate (n = 8)

In the 6 months prior to ATU entry, patients experienced a median (range) of 13.5 (1–99) attacks (mean [SD] of 16.2 [14.6] attacks); 33% of patients experienced 13–24 attacks. Most patients (94%) were previously exposed to LTP (at any point during their lifetime), and the majority (78%) were receiving LTP prior to ATU entry. Almost a third (30%) were receiving LTP with C1-INH monotherapy, five patients were receiving progestin only, 3 were receiving C1-INH and danazol, and 2 were receiving progestins in combination with another LTP.

### Dose changes

The lanadelumab dose was modified for 2 of 69 patients at follow-up visit 1 and for 10 of 39 patients at follow-up visit 2. In most cases, the dosage interval was increased to every 4 weeks (Additional file 3: Table S3). In one patient, the dose interval was decreased to once a week. The reason for this modification was unclear and this was the latest information available for this patient as part of the cATU.

### HAE attacks over time: analyzed population

**Fig. 2:**
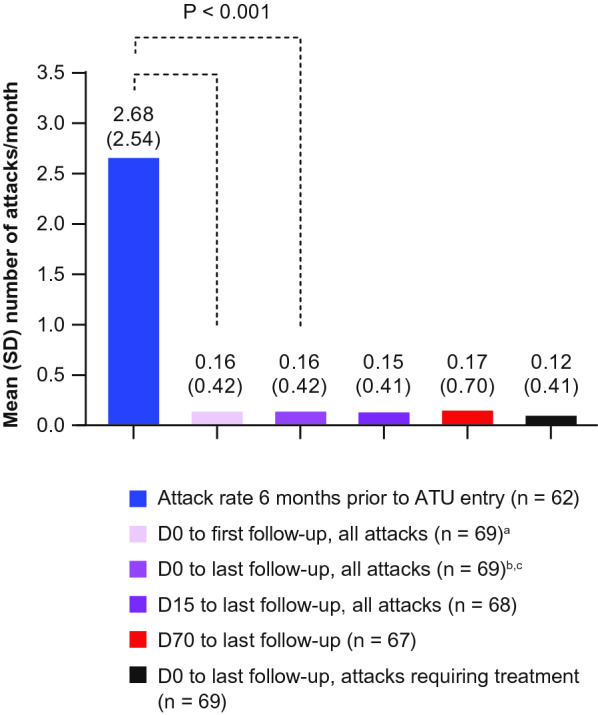
Hereditary angioedema (HAE) attacks (analyzed population). The analyzed population refers to all patients who had ≥ 1 quarterly follow-up visit (n = 69). ^a^Median (range) follow-up duration: 84.0 (61–182) days. ^b^Last follow-up refers to either first or second follow-up visit, whichever occurred prior to the September 23, 2019 data freeze. ^c^Median (range) follow-up duration: 160 (63–232). Last follow-up is the patient’s last visit before September 23, 2019. *ATU* Authorization for Temporary Use, *D* day, *SD* standard deviation

Compared with the mean (SD) number of HAE attacks per month during the 6 month period before ATU entry (2.68 [2.54]), a lower mean (SD) number of attacks occurred between lanadelumab initiation and first follow-up visit (0.16 [0.42]; P < 0.001) or last follow-up visit (either visit 1 or 2, whichever occurred before September 23, 2019; 0.16 [0.42]; P < 0.001); between D15 and the last follow-up visit (0.15 [0.41]); as well as from D70 to the last follow-up visit (0.17 [0.70]). The mean (SD) number of HAE attacks in patients requiring on-demand treatment from D0 until last follow-up (0.12 [0.41]) was also lower compared with the number of attacks prior to ATU entry (Fig. 2).

**Fig. 3:**
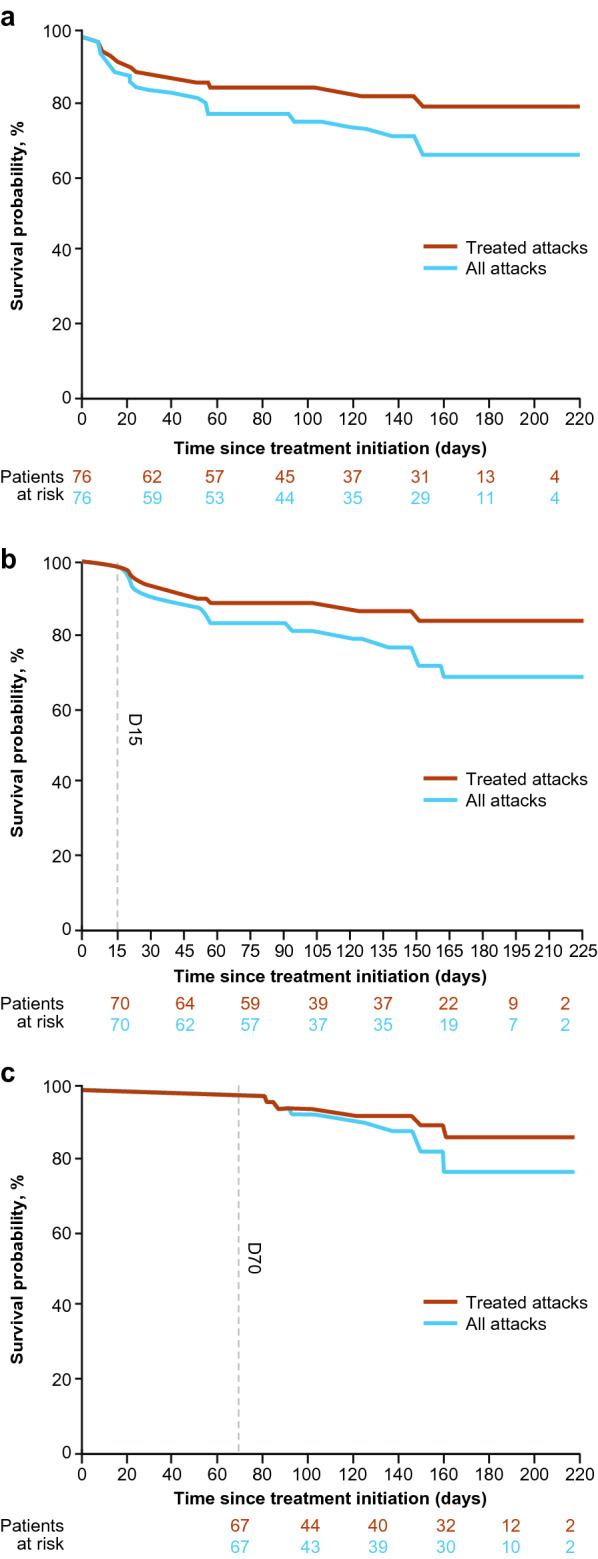
Cumulative percentage of attack-free patients from (**a**) D0 onward, (**b**) D15 onward, and (**c**) D70 onward. *D* day

For all attacks, cumulative percentage of attack-free patients 6 months after treatment initiation was 66% (95% CI 52.1–76.8) from D0 (Fig. 3A), 69% (95% CI 53.8–79.5) from D15 (Fig. 3B), and 77% (95% CI 61.0–87.3) from D70 (Fig. 3C). None of the candidate explanatory variables were significantly associated with HAE attack occurrence after D0.

**Fig. 4:**
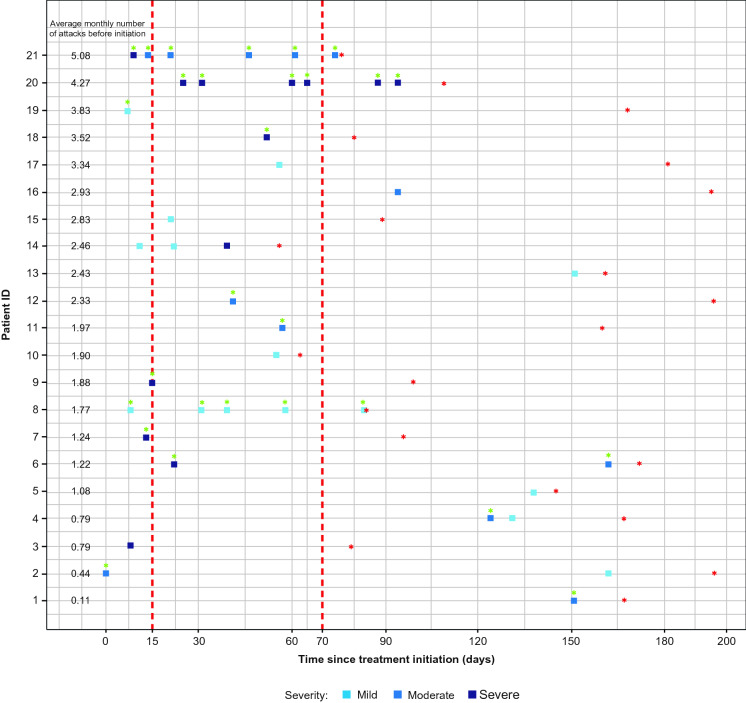
Overview of physician-confirmed hereditary angioedema (HAE) attacks and use of on-demand treatment. Attacks with a green asterisk are treated attacks. The red asterisks correspond to the day of last follow-up for each patient. HAE attack data are reported for 21 of 22 patients who had an attack after D0, as data for attack dates were missing for one patient. All patients who experienced attacks after D0 had received lanadelumab 300 mg every 2 weeks. Although 3 patients underwent dosage modifications, the modifications took place several days after the occurrence of the latest HAE attack. *D* day, *ID* identification

A total of 22 patients experienced an attack after D0; data on the timeline of events were available for 21 patients. A pictorial depiction (based on severity of attacks and whether or not patients received on-demand treatment) is shown in Fig. 4. Respectively, 17 and 10 patients experienced an attack after D15 and after D70. For 6 of these 10 patients, an attack occurred after the steady-state period (D70), but not between D15 and D70. Of note, two patients experienced ≥ 1 attack during all 3 periods—before D15, after D15, and after D70. Their average monthly number of attacks ranged from 1.67 to 5.33 in the 6 months before treatment and from 1.82 to 2.41 throughout the follow-up period. Both patients were receiving ongoing LTP at time of treatment access request.

### AE-QoL asssessments

#### Scores over time

**Fig. 5:**
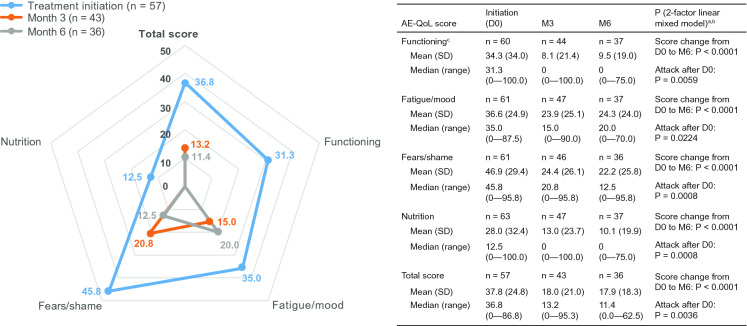
Median Angioedema Quality of Life Questionnaire (AE-QoL) scores. ^a^P-values refer to comparisons of the mean AE-QoL scores shown in this table. ^b^Occurrence of attack after D0 (yes/no) was included as a covariate in this analysis to evaluate whether it had an impact on AE-QoL score change. Nonsignificant (P > 0.05). ^c^This domain reflects impairment in work, physical activity, and social activities [14]. *D* day, *M* month, *SD* standard deviation

Median and mean AE-QoL total scores and scores for each of the AE-QoL domains were significantly higher at treatment initiation compared with months 3 and 6, reflecting QoL improvement (Fig. 5). Per findings from the two-factor linear mixed model analysis, occurrence of an attack after D0 correlated significantly with a worsening QoL for all dimensions except fear/shame. This suggests that whether an attack occurred after D0 did not appear to impact the evolution of feelings of fear or shame over time.

#### Achievement of the MCID

For the AE-QoL total score, 55.3% of patients achieved MCID between D0 and month 3 (n = 38 with available data) and 64.5% of patients between D0 and month 6 (n = 31 patients with available data). The only variable found to be inversely associated with achieving the MCID threshold at month 3 was the number of attacks in the 6 months preceding ATU entry—patients with ≤ 34 attacks were significantly more likely to achieve MCID with lanadelumab treatment than those with > 34 attacks (Additional file 4: Table S4).

### AAS scores

**Fig. 6:**
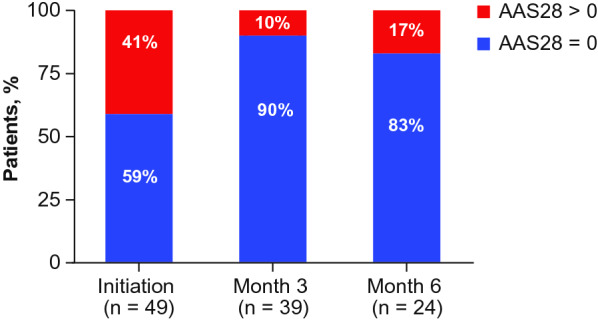
Proportion of patients with 28 day Angioedema Activity Score (AAS28) score of 0 at initiation, month 3, and month 6 (analyzed populaton)

Complete AAS28 scores were available for 49, 39, and 24 patients at D0, month 3, and month 6, respectively. The proportion of patients with an AAS28 score of 0 was higher during month 3 and month 6 of lanadelumab treatment than during treatment initiation, suggesting reduced burden of illness (Fig. 6). For patients with an AAS score > 0, the mean (SD) scores appeared to be similar from D0 to month 6, implying that for patients who continued having HAE attacks after D0, limited improvements in disease severity occurred over the follow-up period.

### Subgroup of patients with ongoing C1-INH LTP prior to lanadelumab treatment initiation

In the total population, 26 patients were receiving C1-INH prior to initiating lanadelumab treatment (IV pdC1-INH concentrate, fixed dose [n = 11]; IV pdC1-INH concentrate, weight based [n = 4]; IV recombinant C1-INH concentrate [n = 8]; C1-INH + oral androgen [n = 3]), and 51 patients were not receiving C1-INH LTP; importantly, all patients discontinued prior LTP upon ATU entry. Patient characteristics were generally similar between these two groups (Additional file 5: Table S5). The only statistically significant difference was a higher mean (SD) age at diagnosis for patients without versus with ongoing C1-INH LTP at ATU entry (10.9 [8.6] vs. 8.4 [4.8], respectively; P < 0.05).

As shown in Additional file 7: Figure S1, lanadelumab appears to be effective in reducing the rate of HAE attacks in patients in the analyzed population who were (n = 24) and who were not (n = 45) receiving ongoing C1-INH therapy at ATU entry. Regardless of prior ongoing C1-INH LTP use, median and mean scores for each AE-QoL domain and, thus, the overall AE-QoL score, improved over time (descriptive data provided in Additional file 8: Figure S2).

### Subgroup of patients with follow-up duration above the median

The median (range) follow-up duration from D0 to the last follow-up (either visit 1 or 2) was 160 (63–232) days. A total of 35 patients (all with HAE-1/2) had a follow-up duration of ≥ 160 days, forming the 6 month follow-up subgroup. HAE attack rates (median [range]) from D0 to last follow-up (0 [0–0.37]), from D15 to last follow-up (0 [0–0.34]), and from D70 to last follow-up (0 [0–0.26]) were similar in this patient subgroup compared with the 69 patients in the full analyzed population (Additional file 6: Table S6).

### Safety (total population)

**Fig. 7:**
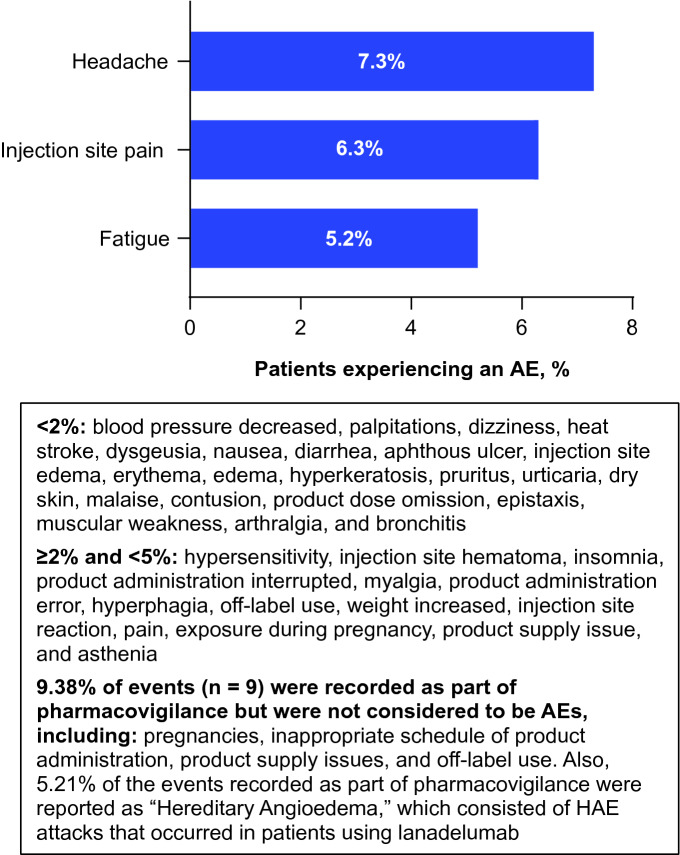
Adverse events (AEs; by Preferred Term) occurring in ≥ 5% frequency in the total population (n = 77). All AE were nonserious. *HAE* hereditary angioedema

During the treatment period, 96 AEs were reported by 46 patients. No serious AEs were reported, and no deaths occurred during the analysis period. Headache and injection site pain were among the most frequently reported AEs (Fig. 7). Five events led to treatment discontinuation, including pregnancy (n = 1), pain or injection site pain (n = 3), and patient decision (n = 1).

### Injection training and injection use satisfaction

At the time of data cut-off, 62 patients had completed a patient diary regarding the ease with which they learned how to inject lanadelumab at D0. The majority of patients (77%) had received a practical demonstration by a nurse or physician, 27% were informed via leaflets, and 10% reported “other” methods (one patient received a verbal explanation by a nurse and four patients already knew how to use an injection).

Most patients (71%, based on n = 54) found their method of learning to be very easy (mean [SD] score 8.8 [2.0]), based on a scale of 1 (very difficult) to 10 (very easy). Overall satisfaction levels regarding ease of lanadelumab administration remained generally consistent throughout the follow-up period. Based on a scale of 1 (not at all satisfied) to 10 (very satisfied), the mean (SD) overall satisfaction level was 8.1 (1.9) at month 1 (injection 1; n = 59) and 8.7 (1.7) at month 6 (injection 2; n = 26).

## Discussion

This analysis of early access to treatment with lanadelumab presents findings of lanadelumab effectiveness, safety, and impact on QoL.

Consistent with efficacy findings in the HELP [12] and HELP OLE studies [19], treatment with lanadelumab (primarily dosed 300 mg every 2 weeks) in this study resulted in lower HAE attack rates compared with rates prior to treatment. Most patients (66%) were attack free through 6 months of treatment. Of note, although the majority of patients reported use of ongoing LTP prior to lanadelumab initiation, the baseline HAE attack rate was high, reflecting unmet needs. These findings are consistent with those reported from a US-based patient survey evaluating burden of disease in patients with HAE-1/2, which was conducted prior to the US Food and Drug Administration approval of lanadelumab or subcutaneous C1-INH [20]. In the current study, lanadelumab was effective regardless of ongoing C1-INH LTP use at ATU entry. The fact that patients were able to successfully switch from their previous LTP to lanadelumab emphasizes its effectiveness.

Exploratory findings in patients with 6 months of follow-up showed similar HAE attack rates compared with those in the full analysis population (for whom follow-up duration was highly variable). Notably, attack rates were lowest from D70 (the approximate time by which steady state is expected to be reached), supporting findings from the HELP Study, in which 77% of patients treated with lanadelumab 300 mg every 2 weeks were attack free during the steady state period (D70–D182), compared with 44% of patients receiving this dose during the full 26 week treatment period [12]. In the current study, 6 months after treatment initiation, the cumulative percentage of attack-free patients after D70 was 77%.

The heavy burden of HAE on patients’ lives is an ongoing challenge [20]; impairments across multiple QoL dimensions are continually reported [7, 21, 22]. In the current study, treatment with lanadelumab resulted in improved QoL, as reflected by a greater proportion of patients with AAS28 scores of 0 after versus before treatment initiation, as well as lower AE-QoL total scores regardless of prior C1-INH LTP use; MCID for the AE-QoL total score was achieved in most patients. These findings corroborate QoL improvements demonstrated with lanadelumab in the HELP Study [23]. In addition, findings showed that at 3 months of treatment, patients with ≤ 34 attacks in the 6 months prior to ATU entry were more likely to achieve MCID than those with > 34 attacks. However, the relatively large 95% confidence interval for the odds ratio indicates that this result should be interpreted with caution owing to its low precision.

Results further showed that occurrence of an HAE attack after D0 correlated significantly with lower QoL improvement in all domains other than fears/shame. This finding underscores the negative impact of HAE on patients’ emotional wellbeing—previous findings have demonstrated a continuing fear of attacks even during attack-free periods [7]. Mean AAS scores among patients with a score > 0 appeared to be similar from D0 through month 6, which implies that limited improvements in disease activity occurred over the follow-up period for patients who continued having HAE attacks after D0.

As previously shown in the HELP [12] and HELP OLE studies [24], lanadelumab was generally well tolerated; no new safety signals were found. Most patients were satisfied with the ease of self-administering lanadelumab injections, reflected by high overall satisfaction ratings at months 1 and 6. These findings are encouraging, as fear of injections has been noted as an important barrier to self-administration of HAE therapies [25].

Several limitations are worth mentioning. Although the study protocol specified that dose adjustments could be made at the physicians’ discretion after ≥ 6 months of treatment, 2 dose changes were made prior to the 6 month visit. Also, the planned schedule for follow-up visits was every 3 months, yet actual time intervals between visits varied. However, the 6 month follow-up analysis subgroup was evaluated in the effort to account for such variability. Also, since this ATU was a compassionate program, rates of missing data were high (especially related to AE-QoL, AAS28, and injection satisfaction assessments), and an open-label study design complicates the interpretation of patient-reported outcome findings. In addition, depending on the date on which lanadelumab was initiated, a substantial proportion of patients had not yet had their 6 month follow-up visit by the September 23, 2019, data cut-off date (this date was based on the required 6 month cut-off period, per the monitoring deadline set by the ASNM protocol), or data for these patients were incomplete. With this in mind, efficacy findings should be assessed within the context of the small number of patients evaluated at the 6 month follow-up visit (n = 39). The short-term results from this cATU represent the first experience with lanadelumab in France. The 77 patients who received a first dose represent approximately 26% of the estimated 300 people with HAE in France who would be eligible to use lanadelumab based on an estimate from the French HAS. An observational real-world post-ATU cohort study, SERENITI, is underway in France, which aims to evaluate the effectiveness and safety of lanadelumab in patients who received or will receive ≥ 1 dose of lanadelumab after October 2018. This 3 year study began recruiting patients in December 2019, most of whom participated in the ATU, and is expected to end in April 2024 [26].

## Conclusions

In this analysis, lanadelumab (as the sole prophylactic agent) effectively reduced the HAE attack rate, improved QoL, and was generally well tolerated. These results and patient experiences confirm those demonstrated in the pivotal HELP Study and HELP OLE, thus helping to establish the effectiveness and safety of lanadelumab in the prevention of HAE attacks. In addition, most patients were satisfied with the ease of self-administration of lanadelumab. As the armamentarium of targeted treatments grows, the goals of therapy and models of care for HAE are shifting toward acheivement of complete control of disease (i.e., attack-free periods), with minimal impact of disease on QoL. The positive findings from the current study show further support that lanadelumab can help achieve these goals.

## Supplementary Information

**Additional file 1: Table S1.** Schedule of assessments.

**Additional file 2: Table S2.** Explanatory variables for the multivariate logistic regression analyses.

**Additional file 3: Table S3.** Lanadelumab dose modifications.

**Additional file 4: Table S4.** Odds ratio for baseline variables associated with final AE-QoL score decrease below the MCID threshold, from D0 to M3 (n = 38).

**Additional file 5: Table S5.** Baseline characteristics based on ongoing C1-INH LTP before lanadelumab initiation.

**Additional file 6: Table S6.** Hereditary angioedema attacks in the analyzed population and in the subgroup of patients with follow-up duration above the median.

**Additional file 7: Figure S1.** Hereditary angioedema attack rates based on ongoing use of C1 inhibitor (C1-INH) long-term prophylaxis (LTP) before lanadelumab initiation in the total population. **a** Ongoing C1-INH LTP before D0; **b** no ongoing C1-INH LTP before D0. Last follow-up is the patient’s last visit before September 23, 2019 *D* day, *SD* standard deviation.

**Additional file 8: Figure S2.** Angioedema Quality of Life Questionnaire (AE-QoL) score based on ongoing use of C1 inhibitor (C1-INH) long-term prophylaxis (LTP) at lanadelumab initiation. **a** Ongoing C1-INH LTP before D0; **b** no ongoing C1-INH LTP before D0 *D* day, *SD* standard deviation.

## Data Availability

The data sets generated and/or analyzed during the current study are available from the corresponding author on reasonable request to researchers who provide a methodologically sound proposal. Data will be provided after de-identification to ensure protection of the personal data of patients participating in the study.

## Abbreviations

AAS: Angioedema Activity Score
AAS28: 28-day AAS
AE: Adverse event
AE-QoL: Angioedema Quality of Life questionnaire
ANSM: Agence Nationale de Sécurité du Médicament et des produits de santé (French National Agency for Medicines and Health Products Safety)
ATU: Authorization for temporary use
cATU: ATU in a cohort
C1-INH: C1 inhibitor
D: Day
HAE: Hereditary angioedema
HAE-1/2: HAE type 1 or 2
OLE: Open-label extension
IV: Intravenous
LTP: Long-term prophylaxis
MCID: Minimal clinically important difference
nATU: Nominative ATU
Pd: Plasma-derived
SD: Standard deviation
