# Supplementary material for: Long-term prophylaxis with lanadelumab for HAE: authorization for temporary use in France
Source: Allergy Asthma Clin Immunol. 2022 Apr 1;18:30. doi: 10.1186/s13223-022-00664-4 (PMC8976389; doi:10.1186/s13223-022-00664-4)
Supplement: Supplementary file 1 — Additional file 1: Table S1. Schedule of assessments. [file 13223_2022_664_MOESM1_ESM.docx]

| **Additional file 1: Table S1.** Schedule of assessments | | | | |
| --- | --- | --- | --- | --- |
|  | Visit | | | |
| Evaluation/examinations | Treatment access request | Treatment initiation (D0 visit) | Follow-up visits (every 3 months) | Treatment discontinuation |
| Data assessed by the physician | | | | |
| Patient consent (via telephone or on-site visits) and information | X | X |  |  |
| Verification of indication and contraindications | X |  |  |  |
| Diagnosis/laboratory findings | X^a^ |  |  |  |
| Disease history | X |  |  |  |
| Baseline blood tests | X^a^ | X^b^ |  |  |
| Prior prophylaxis and treatments for acute HAE attacks | X |  |  |  |
| Prescribed dosage |  | X | X |  |
| Prescribed concomitant treatments |  | X | X |  |
| Discontinuation of lanadelumab (and cause for discontinuation) |  |  |  | X |
| Occurrence of attacks (number/site/severity/ treatment taken) | X | X | X | X |
| Occurrence of any adverse event (and pregnancy or pregnancy planning) | X | X | X | X |
| Data reported by the patient | | | | |
| Self-injection training/ease of learning |  | X |  |  |
| Adherence to treatment/ease of use and level of satisfaction |  |  | X | X |
| AAS28 |  | X | X | X |
| AE-QoL |  | X | X | X |

*AAS28* 28-day Angioedema Activity Score, *AE-QoL* Angioedema Quality of Life questionnaire, *D* day, *HAE* hereditary angioedema

^a^ Based on patient medical records

^b^ If no tests have been performed
