# Supplementary material for: Long-term prophylaxis with lanadelumab for HAE: authorization for temporary use in France
Source: Allergy Asthma Clin Immunol. 2022 Apr 1;18:30. doi: 10.1186/s13223-022-00664-4 (PMC8976389; doi:10.1186/s13223-022-00664-4)
Supplement: Supplementary file 2 — Additional file 2: Table S2. Explanatory variables for the multivariate logistic regression analyses. [file 13223_2022_664_MOESM2_ESM.docx]

| **Additional file 2: Table S2.** Explanatory variables for the multivariate logistic regression analyses | | |
| --- | --- | --- |
| Outcome assessed | HAE attacks^a^ | AE-QoL scores |
| HAE type (1 or 2) | X | X |
| Age at ATU entry (continuous variable) | X | X |
| Sex (male or female) | X | X |
| Time elapsed since diagnosis (continuous variable) | X | X |
| Laryngeal attack history (yes or no) | X | X |
| Occurrence of ≥ 1 severe attack before D0 (yes or no) | X | X |
| Baseline AE-QoL total score (continuous variable) | X |  |
| Number of attacks 6 months before ATU entry (lower vs. above the median) | X |  |
| Latest LTP treatment before D0 (e.g., none, C1-INH, oral, multitherapy) | X | X |
| Number of attacks 6 months before ATU entry (terciles: < 25, 25–34, > 34) |  | X |
| Occurrence of all and treated HAE attacks (yes or no) after D0 and D15 for scores at M3, and after D0, D15, and D70 for scores at M6 |  | X |
| Secondary nonresponse (yes or no; defined as patients receiving lanadelumab who did not experience an attack between D15 and D70 but did so any time after D70) |  | X |

*AE-QoL* Angioedema Quality of Life questionnaire, *ATU* Authorization for Temporary Use, *C1-INH* C1 inhibitor*, D* day, *HAE* hereditary angioedema, *LTP* long-term prophylaxis, *M* month

^a^ For the analysis of HAE attacks: Continuous explanatory variables were first categorized in terciles to secure number of patients in each category and to assess linearity of the association with dependent variables. Cutoffs associated with tercile categorization were assessed for clinical relevance and adjusted, as possible; Variables were only included in the model if they were associated with attack occurrence in univariate analyses, with P < 0.20. Variables were then entered into the multivariate model via forward stepwise introduction using a 0.05 significance level
