# Supplementary material for: Long-term prophylaxis with lanadelumab for HAE: authorization for temporary use in France
Source: Allergy Asthma Clin Immunol. 2022 Apr 1;18:30. doi: 10.1186/s13223-022-00664-4 (PMC8976389; doi:10.1186/s13223-022-00664-4)
Supplement: Supplementary file 3 — Additional file 3: Table S3. Lanadelumab dose modifications. [file 13223_2022_664_MOESM3_ESM.docx]

| **Additional file 3: Table S3.** Lanadelumab dose modifications | | | |
| --- | --- | --- | --- |
| Patient | Duration between D0 and the visit when dose was modified | New dosing frequency (lanadelumab 300 mg)^a^ | Reason for modification |
| Patients who underwent a dose modification at the first follow-up visit | | | |
| Patient 1 | 92 days | Every 4 weeks | Soreness/aches |
| Patient 2 | 92 days | Every 4 weeks | Pain during injection/ patient request |
| Patients who underwent a dose modification at the second follow-up visit | | | |
| Patient 3 | 196 days | Every 4 weeks | Asthenia and myalgia |
| Patient 4 | 191 days | Every 3 weeks | Pain during injection and no attack |
| Patient 5 | 210 days | Every 1 week | Ease of obtaining product from hospital pharmacy *(reason not clear)* |
| Patient 6 | 198 days | Every 4 weeks | No attack/well tolerated |
| Patient 7 | 164 days | Every 4 weeks | Trial of treatment spacing |
| Patient 8 | 164 days | Every 4 weeks | No attack at 6 months |
| Patient 9 | 192 days | Every 4 weeks | Good progress |
| Patient 10 | 195 days | Every 4 weeks | Spacing of treatment |
| Patient 11 | 163 days | Every 3 weeks | Patient request |
| Patient 12 | 168 days | Every 3 weeks | Patient request |

*D* day

^a^ All patients were prescribed 300 mg lanadelumab every 2 weeks at initiation
