# Supplementary material for: Long-term prophylaxis with lanadelumab for HAE: authorization for temporary use in France
Source: Allergy Asthma Clin Immunol. 2022 Apr 1;18:30. doi: 10.1186/s13223-022-00664-4 (PMC8976389; doi:10.1186/s13223-022-00664-4)
Supplement: Supplementary file 4 — Additional file 4: Table S4. Odds ratio for baseline variables associated with final AE-QoL score decrease below the MCID threshold, from D0 to M3 (n = 38). [file 13223_2022_664_MOESM4_ESM.docx]

| **Additional file 4: Table S4.** Odds ratio for baseline variables associated with final AE-QoL score decrease below the MCID threshold, from D0 to M3 (n = 38) | | | | |
| --- | --- | --- | --- | --- |
| Explanatory variable | Modality (condition 1 vs. condition 2) | Odds ratio | 95% CI | P (χ^2^) |
| Number of attacks in the 6 months prior to ATU entry | Patients with < 25 attacks vs. patients with > 34 attacks | 9.000 | 1.140–71.037 | P = 0.0210 |
|  | Patients with 25–34 attacks vs. patients with > 34 attacks | 13.499 | 1.802–101.119 |  |

*AE-QoL* Angioedema Quality of Life Questionnaire, *ATU* Authorization for Temporary Use, *CI* confidence interval, *D* day, *M* month, *MCID* minimal clinically important difference
