# Supplementary material for: Long-term prophylaxis with lanadelumab for HAE: authorization for temporary use in France
Source: Allergy Asthma Clin Immunol. 2022 Apr 1;18:30. doi: 10.1186/s13223-022-00664-4 (PMC8976389; doi:10.1186/s13223-022-00664-4)
Supplement: Supplementary file 5 — Additional file 5: Table S5. Baseline characteristics based on ongoing C1-INH LTP before lanadelumab initiation. [file 13223_2022_664_MOESM5_ESM.docx]

| **Additional file 5: Table S5.** Baseline characteristics based on ongoing C1-INH LTP before lanadelumab initiation | | |
| --- | --- | --- |
| Characteristic | Prior C1-INH LTP (n = 26) | No prior C1-INH LTP  (n = 51) |
| Median (range) age, years | 37.2 (17.5–68.6) | 44.1 (11.7–78.9) |
| Age, years, n (%) |  |  |
| < 18 | 1 (3.8) | 5 (9.8) |
| 18–64 | 23 (88.5) | 40 (78.4) |
| ≥ 65 | 2 (7.7) | 6 (11.8) |
| Female, n (%) | 17 (65.4) | 36 (70.6) |
| Mean (SD) weight, kg | 79.0 (20.9) | 74.2 (23.8) |
| Mean (SD) age at diagnosis, years | 8.4 (4.8)^a^ | 10.9 (8.6)^a^ |
| HAE type |  |  |
| 1 | 21 (80.8) | 47 (92.2) |
| 2 | 5 (19.2) | 2 (3.9) |
| Normal C1-INH (Type III) | 0 | 1 (2.0) |
| Acquired angioedema | 0 | 1 (2.0) |
| Laryngeal attacks for the 3 most severe attacks in the 6 months prior to ATU entry, n (%) |  |  |
| Number of severe attacks | 42 | 109 |
| Pharyngo-laryngeal attacks | 2 (4.8) | 10 (9.7) |
| Time since most recent attack prior to ATU entry, n (%) |  |  |
| < 6 months | 21 (80.8) | 50 (98.0) |
| ≥ 6 months | 5 (19.2) | 1 (2.0) |
| HAE attacks in the 6 months prior to ATU entry |  |  |
| Number of attacks | 20 | 50 |
| Median (range)^b^ | 13.5 (1–99) | 13.5 (1–40) |

*ATU* Authorization for Temporary Use, *C1-INH* C1 inhibitor, *HAE* hereditary angioedema, *LTP* long-term prophylaxis, *SD* standard deviation

^a^ P < 0.05

**^b^** Physician-confirmed HAE attack data was incomplete for seven patients prior to ATU entry. As such, those patients were excluded at baseline for calculation of HAE attacks before ATU entry
