# Supplementary material for: Long-term prophylaxis with lanadelumab for HAE: authorization for temporary use in France
Source: Allergy Asthma Clin Immunol. 2022 Apr 1;18:30. doi: 10.1186/s13223-022-00664-4 (PMC8976389; doi:10.1186/s13223-022-00664-4)
Supplement: Supplementary file 6 — Additional file 6: Table S6. Hereditary angioedema attacks in the analyzed population and in the subgroup of patients with follow-up duration above the median. [file 13223_2022_664_MOESM6_ESM.docx]

| **Additional file 6: Table S6.** Hereditary angioedema attacks in the analyzed population and in the subgroup of patients with follow-up duration above the median | | | |
| --- | --- | --- | --- |
| Number of attacks per month, median (range) | Time period | | |
|  | D0 to last follow-up | D15 to last follow-up | D70 to last follow-up |
| Subgroup of patients with follow-up duration above the median | 0 (0–0.37)  (n = 34) | 0 (0–0.34)  (n = 33) | 0 (0–0.26)  (n = 33) |
| Analyzed population | 0 (0–2.41)  (n = 69) | 0 (0–2.0)  (n = 68) | 0 (0–5.08)  (n = 67) |

*D* day
